# Short Review on Predicting Fouling in RO Desalination

**DOI:** 10.3390/membranes7040062

**Published:** 2017-10-24

**Authors:** Alejandro Ruiz-García, Noemi Melián-Martel, Ignacio Nuez

**Affiliations:** 1 Department of Mechanical Engineering, University of Las Palmas de Gran Canaria, Las Palmas de Gran Canaria 35017, Spain; 2 Department of Process Engineering, University of Las Palmas de Gran Canaria, Las Palmas de Gran Canaria 35017, Spain; noemi.melian@ulpgc.es; 3 Department of Electronic and Automatic Engineering, University of Las Palmas de Gran Canaria, Las Palmas de Gran Canaria 35017, Spain; ignacio.nuez@ulpgc.es

**Keywords:** reverse osmosis, membrane fouling, fouling indices, predicting models

## Abstract

Reverse Osmosis (RO) membrane fouling is one of the main challenges that membrane manufactures, the scientific community and industry professionals have to deal with. The consequences of this inevitable phenomenon have a negative effect on the performance of the desalination system. Predicting fouling in RO systems is key to evaluating the long-term operating conditions and costs. Much research has been done on fouling indices, methods, techniques and prediction models to estimate the influence of fouling on the performance of RO systems. This paper offers a short review evaluating the state of industry knowledge in the development of fouling indices and models in membrane systems for desalination in terms of use and applicability. Despite major efforts in this field, there are gaps in terms of effective methods and models for the estimation of fouling in full-scale RO desalination plants. In existing models applied to full-scale RO desalination plants, neither the spacer geometry of membranes, nor the efficiency and frequency of chemical cleanings are considered.

## 1. Introduction

Despite improvements and advances in our knowledge of water desalination, one of the main challenges of membrane technology, particularly in Reverse Osmosis (RO) technology, has been how to deal with membrane fouling [1,2,3].

Membrane fouling results from the accumulation of undesirable materials on, in or near the membrane and involves one or more of the following types [3,4]: (a) particulate and colloidal matter deposition on the membrane surface [5]; (b) organic fouling [6]; (c) scaling and inorganic fouling [7]; and (d) biofouling due to adhesion and bacterial growth on the surface of the membrane generating a layer of gel [8].

The consequences of this inevitable phenomenon have a negative effect on the performance of the desalination system (decline in water production over time for constant pressure operations or an increase in required feed) that requires costly pretreatment, higher operating pressures and frequent chemical cleanings, which can damage membranes, degrade permeate quality, and hasten membrane replacement. This additionally increases water cost and energy consumption [9,10]. Therefore, one of the most important challenges is to understand the factors involved in membrane fouling and the subsequent reduction of permeate flux or applied pressure increase that is inevitably associated with membrane processes.

A great deal of research has been carried out to this field in the last 30 years, and although desalination technology is being extensively studied, much remains to be done and researched in the field of membrane fouling. Research that has been undertaken focuses on six key areas: (1) characterization of foulant agents by autopsy studies of membrane elements; (2) understanding of fouling mechanisms; (3) indices for predicting fouling; (4) modeling for full-scale systems; (5) optimization of pre-treatment and chemical cleaning; and (6) optimizing the membrane material and enhanced module design. The first four areas attempt to address directly how fouling occurs and how to predict it, while the others focus more on the mitigation and prevention of fouling, as for example through the use of antifouling membranes [11,12,13,14,15].

Focusing on attempts to address directly and predict model membrane fouling, several fouling prediction tools and techniques have been developed to describe membrane fouling [16,17,18,19,20]. The traditional and most widely-applied fouling indices in RO systems are the Silt Density Index (SDI) and the Modified Fouling Index (MFI). However, these indices have a limitation in predicting RO fouling rate such as lack of precision with small (<0.45 μm) foulant agents [21,22,23].

Some recent research has focused on modifying these methods and using membranes more similar to those of RO in order to evaluate fouling potential [17,22,24,25], while another research focus is the proposal of prediction models based on experience in full-scale RO desalination plants [26,27,28,29].

This paper provides a critical review evaluating the state of industry knowledge in the development of fouling indices and models in membrane systems for desalination in terms of use and applicability.

## 2. Membrane Fouling Indices

The Silt Density Index (SDI) and Modified Fouling Index (MFI) are common parameters or indices to determine the fouling potential (mainly colloidal) of feedwaters in RO systems. Microfiltration (MF) membranes with a pore size of 0.45 μm, which is larger by several orders of magnitude than the pore size of the RO membranes, are used to calculate these indices. Although these indices were developed to evaluate RO membrane fouling, they can also serve as a reference in the evaluation of fouling in porous membranes like MF and Ultrafiltration (UF).

These indices are based on conventional and dead-end filtrations, while commercial applications are performed in cross-flow filtration. This implies that the flow conditions in the module are not taken into account, though this is a crucial parameter in the optimization of the process. However, the experimental determination of these data is very simple and frequently used.

### 2.1. Silt Density Index

SDI is used to predict the colloidal fouling potential of feedwaters in RO systems and the efficiency of pre-treatments. SDI measurement is performed using the standard ASTM D4189 [30]. The feedwater is filtrated in dead-end mode by an MF membrane with a diameter of 47 mm and a pore size of 0.45 μm at a constant pressure of 207 kPa (30 psi). The two time intervals measured at the beginning of filtration are the initial ($t_{i}$) and final ($t_{f}$) time to collect 500 mL of permeate, respectively. The third time interval (*t*) can be 5, 10 or 15 min, which is the period between $t_{i}$ and $t_{f}$. SDI is calculated by the following Equation (1):

(1) $$SDI=\frac{1-\frac{t_{i}}{t_{f}}}{t}\cdot 100$$

Generally, membrane manufactures suggest a value below three for SDI, but four or five are also acceptable values. Most pre-treatment studies are based on $SDI_{15}<3$. Standard ASTM D4189 [30] specifies that the membranes must have a mean pore size of 0.45±0.2 μm, and the values of SDI obtained with membranes of different suppliers, which present differences in their morphology (porosity, for example), may differ.

SDI has its limitations, and a lack of reliability has been demonstrated in several studies [31,32,33]. SDI is a static measurement of resistance assuming lineal permeate flux decline. This allows good results to be obtained when the water has a high quality, as the initial and final fluxes would be similar. However, the use of SDI may not be appropriate when the water has a high fouling potential, since SDI has no linear relation with the colloidal content. In this case, derivation of this index is very empirical and is not based on any mechanisms of fouling [31,34]. For these reasons, SDI should not be used as input in the mathematical model to predict fouling rates [35]. To overcome the limitations of SDI, J.C. Schippers and J. Verdouw [31] proposed a different parameter: the Membrane Fouling Index (MFI).

### 2.2. Modified Fouling Index

MFI (also called $MFI_{0.45}$) is a parameter based on the filtration mechanism of layer deposition or cake formation and takes into account the mechanism of the reduction of flow that takes place in membrane systems. Therefore, it better represents the operating conditions of the membranes than SDI and can be used to measure water with a high and low fouling potential.

MFI [36] is determined using similar equipment and procedures as SDI, except that the volume of permeate water is measured in 30-s intervals over 15 min of filtration. In this period, the data of permeate volume and *t* are collected. A better understanding of the experimental data that are obtained is achieved by using Equation (2) as proposed by J.C. Schippers and J. Verdouw [31]. Equation (2) is based on the resistances-in-series model and considers that fouling resistance is due to cake formation on membrane surfaces. Equation (2) shows a lineal relation between t/V (s/L) and *V* (L). The slope of this equation is the value of MFI (Equation (3)).

(2) $$\frac{t}{V}=\frac{\mu \cdot R_{m}}{\Delta p\cdot A}+\frac{\mu \cdot \alpha \cdot C_{b}}{2\cdot \Delta p\cdot A^{2}}\cdot V$$

(3) $$MFI=\frac{\mu \cdot \alpha \cdot C_{b}}{2\cdot \Delta p\cdot A^{2}}$$

where Δp (Pa) is the transmembrane pressure, μ (Pa s) is the water viscosity, $R_{m}$ (m${}^{-1}$) is the hydraulic resistance of the membranes, α (m/kg) is the specific resistance of the cake, *A* (m${}^{2}$) is the membrane surface, *V* (L) is the volume and $C_{b}$ (kg/m${}^{3}$) is the concentration of particles in feedwater.

**Figure 1:**
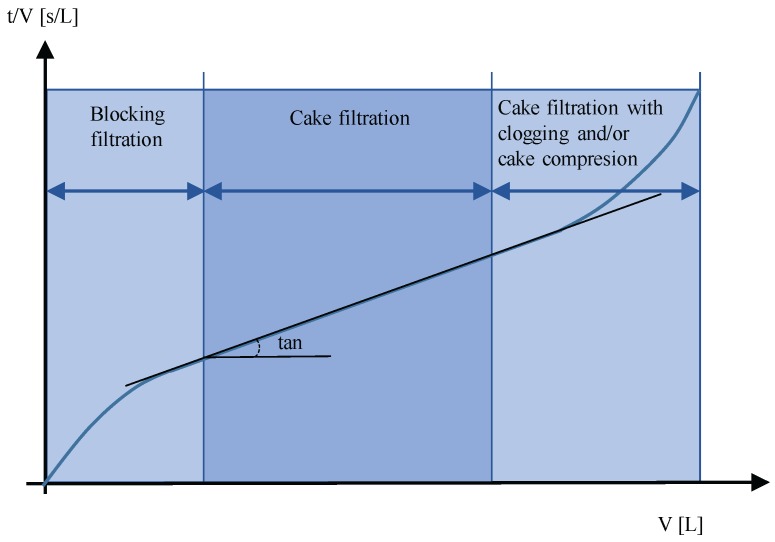
Ratio of filtration time and filtrate volume (t/V) as a function of filtrate volume (*V*) [38]. Copyright Elsevier, 2012.

MFI is determined in the second region of the curve t/V vs. *V* (Figure 1). It can be divided into three stages: blocking filtration, cake filtration (lineal) and cake filtration with clogging and/or cake compressure. In case of a high concentration of colloids, the graph t/V vs. *V* has non-linear behavior throughout the entire period, so MFI is calculated from the first region [37].

Membrane manufacturers suggest using MFI< 1 s/L${}^{2}$ and a maximum value of 4 s/L${}^{2}$ to control membrane fouling. Most studies have been based on a target value less than 1 s/L${}^{2}$. In practice, the calculation of MFI is complex, so in most cases, SDI is calculated. Some recent research has focused on modifying these methods in order to study the applicability of multiple MFIs to evaluate the fouling potential of feed water in a full-scale RO plant [22].

The term $\alpha \cdot C_{b}$ is usually called fouling index *I*. If α and $C_{b}$ are known, *I* can be calculated using Equation (4) [6,39,40,41]:

(4) $$I=\alpha \cdot C_{b}$$

Following the theory of cake deposition or formation, when there is no compaction, the value of cake resistance is $R_{c}$. It can be rewritten as Equation (5) [31,39]:

(5) $$R_{c}=\frac{I\cdot V}{A}=\frac{\alpha \cdot C_{b}\cdot V}{A}$$

The index *I* is related to MFI [31] with the parameters α and $C_{b}$ (Equation (6)):

(6) $$MFI=\frac{\mu \cdot I}{2\cdot \Delta p\cdot A^{2}}$$

MFI depends on the operating conditions of the filtration, Δp and *A* according to Equation (6). A normalization in the same condition as SDI is required. Otherwise, *I* does not depend on operating conditions, so the parameter α does not vary as a result of the effect of cake compressibility. It can be considered that *I* is already a normalized value of MFI, which depends on pressure and membrane surface (Equation (6)). However, values of MFI under different conditions of filtration with the same water sample are not the same as for *I* [42]. Equation (5) is rewritten as follows:

(7) $$I=\frac{R_{c}}{V/A}$$

The fouling index can be interpreted as a fouling parameter referring to the increase in cake resistance ($R_{c}$) divided by the specific permeate volume (V/A) (by cake formation as the only type of fouling mechanism).

The value of $R_{c}$ of the deposited foulants on the membrane surface can be calculated knowing *I* and $C_{b}$ (Equation (6)). However, the specific resistance of the cake (or permeability of the cake) is affected by the pressure applied, and that effect can be represented (as a first approximation) by an empirical expression in the form of Equation (8) [43].

(8) $$\alpha =\alpha_{0}\cdot \Delta p^{n}$$

where $\alpha_{0}$ is the cake-specific resistance at reference pressure and Δp is the pressure gradient working with the reference pressure. *n* is the compressibility coefficient. The effects of pressure and compressibility on the characteristics of the cake and colloidal dispersion is a complicated topic that is still under investigation.

Index *I* is defined by Equation (4), and its value is calculated by the experimental determination of MFI (Equation (6)). The parameter *I* is related to the fouling potential of feedwater, which is defined by multiplication of two characteristics: its specific resistance α and concentration $C_{b}$.

### 2.3. Indices Derived from SDI

A. Alhadini et al. [44] proposed a normalized SDI ($SDI^{+}$). This index takes into consideration the temperature (*T*), Δp, $R_{m}$ and different fouling mechanisms by using a line chart assuming cake filtration and 100% particle retention. In the same work, they proposed the volume-based SDI ($SDI_{v}$). This fouling index compares the initial flow rate with the flow rate after the filtration of the standard volume. $SDI_{v}$ has a linear relationship with the particle concentration if complete blocking is the dominant fouling mechanism in the test. They concluded that $SDI_{v}$ is a better index to estimate the fouling potential of feedwater in RO than SDI.

### 2.4. Indices Derived from MFI

J.C. Schippers and J. Verdouw [31] showed that MFI depends on the membrane molecular weight cut-off. Few authors have developed procedures to calculate MFI using membranes with smaller pore size. Table 1 shows a summary of procedures for calculating MFI, as well as the indices, parameters and methods used to measure the fouling potential. The advantages and disadvantages of each procedure have been commented on in works referenced in Table 1 and others [38,45].

S.F.E. Boerlage et al. [35] showed that the MF membrane (0.45 μm) used for MFI was not suitable for fouling of small size colloids. This fouling can happen in RO membranes if the pre-treatment does not separate these particles. The same authors [35] developed MFI−UF at constant pressure ($MFI-UF_{\mathrm{const}.\;\mathrm{pressure}}$). This procedure uses a UF membrane instead of an MF membrane to separate more particles, but it can take more than 20 h.

The aforementioned fouling indices have been measured at constant pressure, whereas most membrane systems works at constant flux. S.F.E. Boerlage et al. [46] further developed the $MFI-UF_{\mathrm{const}.\;\mathrm{pressure}}$ in order to adapt it to constant flux conditions. This resulted in a noticeable difference in the duration of the test compared to MFI−UF at constant pressure; MFI−UF at constant flux ($MFI-UF_{\mathrm{cont}.\;\mathrm{flux}}$) could be obtained in 2 h.

Recently, S. Khirani et al. [42] proposed NF−MFI using a Nanofiltration (NF) membrane to measure MFI. As is shown in Table 1, the NF−MFI is measured at constant pressure. Khirani et al. [42] showed that fouling potential could be measured by the NF−MFI, even for small organic particles. Although this method is a step towards obtaining more realistic fouling indices, the mode of operation was still at constant pressure and dead-end flow.

**Figure 2:**
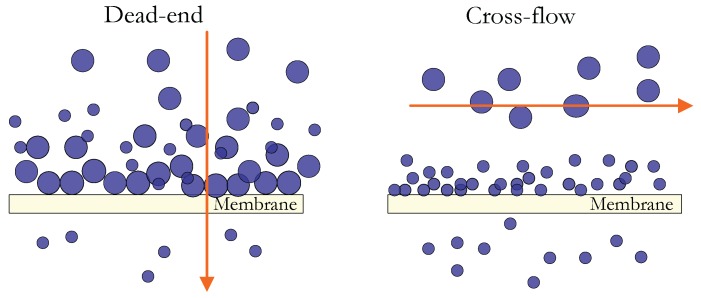
Filtration modes: dead-end and cross-flow.

Modified methods for measuring MFI have the disadvantage that they require a long measuring time with more complex systems than SDI or MFI itself. The filtration mode is dead-end flow, so it is not close to real conditions in terms of hydrodynamic flux in RO process. Cross-flow hydrodynamic conditions influence the selective deposition of smaller particles or colloids, which are the most likely to be deposited on membranes, as illustrated in Figure 2.

Due to the balance between the convection flow and the backscattering of particles, the larger particles with higher backscattering speeds tend to move away from the surface of the membrane, whereas the smaller particles are preferably deposited as soiling agents. These cross-flow hydrodynamic conditions lead to a different composition and structure of the cake when compared to the final blind filtration [47].

Figure 2 shows the cross-flow filtration. All foulants in the feedwater are deposited or passed through the membrane, as in the case of the measurements of SDI, MFI and MFI−UF, while in cross-flow filtration, foulants are fractionated by selective deposition. These hydrodynamic effects could lead to inaccuracies in the extension of SDI and MFI that is performed in dead-end flow.

To take into consideration, the effect of small particles or colloids in MFI, S.S. Adham and A.G. Fane [48] proposed the use of a selective MF membrane to be operated in cross-flow mode. They called this index the Cross-Flow Sampler-MFI (CFS−MFI). After MF membrane filtration (colloid matter passes though this membrane), MFI/SDI is measured as shown in Table 1. Although this method is a better approach, the cross-flow MF is separated from the measurement device in dead-end flow, so CFS−MFI is determined in discontinued mode.

M.A. Jaaved et al. [49] calculated CFS−MFI in continuous operation mode with MFI (in dead-end flow) directly connected to CFS. Recently, L.N. Sim et al. [50] applied CFS to $MFI-UF_{\mathrm{cont}.}$ to simulate selective colloidal deposition in real RO systems. The proposed index is known as $CFS-MFI_{\mathrm{UF}}$ and uses a UF membrane for MFI. The particles that pass across CFS and that are deposited on the UF membrane will foul the RO membranes. In a later work, L.N. Sim et al. [51] combined $CFS-MFI_{\mathrm{UF}}$ with a Cake-Enhanced Osmotic Pressure (CEOP) [52] model to predict the cross-flow RO fouling profile under constant flux filtration. They concluded that incorporating the CEOP effect was a very promising method in predicting colloidal fouling in RO.

J. Choi et al. [53] proposed procedures for measuring MFI with different types of membranes (Table 1). The test was called the Combination Fouling Index-MFI (CFI−MFI). It takes into account various foulant agents separated by different membranes. However, the proposed approach is not simple since several types of membranes are required. Although the different measurement systems of MFI improve the prediction of fouling in RO membranes, they are complex and require long times to be determined.

An evaluation of membrane fouling potential by Multiple Membrane Array System (MMAS) was proposed by Y. Yu et al. [54]. MF, UF and NF membranes were connected in series to calculate three MFI values, particle-MFI, colloid-MFI and organic-MFI. MMAS allows the simultaneous separation of target foulants from the feed water and the evaluation of fouling potential of the feed water focusing on the target foulants. The authors suggested that the MMAS could give valuable information about the best candidates for pretreatment and the fouling influence on full-scale RO desalination plants.

M. W. Naceur [55] determined the Dimensionless Fouling Index (DFI). It can be interpreted as the ratio of the membrane resistance to that of the cake due to the concentration of feedwater. The authors mentioned that this index requires performing experiments under different operating conditions to be properly validated.

### 2.5. Fouling Potential Parameter ($k_{\mathrm{fp}}$)

L. Song et al. [59] defined a new standardization method for the determination of fouling potential in membrane processes. Initially, it was developed to evaluate the potential of colloidal fouling in UF membranes, but later was also applied in the characterization of fouling in large-scale RO processes [62,63,64].

Index $k_{\mathrm{fp}}$ (Pa s/m${}^{2}$) (called the fouling potential) is defined by Equation (9):

(9) $$R_{t}=R_{0}+k_{\mathrm{fp}}\cdot \int_{0}^{t}J\;dt$$

In Equation (9), J (m/s) is the specific permeate flux, and *R* and $R_{t}$ (Pa s/m) are the initial and final resistance of the membrane $R_{0}$. In this resistance, the resistive effect of the viscosity is included and is equivalent to multiplication of the resistance as is usually considered, *R* (m${}^{-1}$), and the dynamic viscosity of the fluid μm (Pa s) (Equation (10)):

(10) $$R_{t}=\mu \cdot R$$

If the parameter $k_{\mathrm{fp}}$ is assumed constant over time, it can be calculated using Equation (11):

(11) $$k_{\mathrm{fp}}=\frac{R_{t}-R_{0}}{v_{t}}$$

(12) $$v_{t}=\int_{0}^{t}J\;dt$$

where $v_{t}$ is the total specific volume of permeate over time *t*.

## 3. Predictive Models

These models are an alternative to fouling indices in the prediction of the fouling influence on RO systems. Some authors [26,27,28,29] have proposed equations to estimate the decline of the permeate flux ($J_{w}$) over time due to long-term variation of the water permeability coefficient (*A*). Generally, these correlations are applicable for the respective membrane type and for specific operating conditions.

One of the main drawbacks in the development of this type of model is the availability of long-term operating data for a wide range of operating conditions and different types of full-scale membranes. All models aim to describe the permeate flow decline over time or the variation of the normalized water permeability coefficient $A_{n}$ due to compaction, fouling, etc.

A proposed model to predict the decline of $J_{w}$ due to membrane compaction was used by M. Wilf et al. [26] to estimate the $J_{w}$ decline in the long term (Equation (13)). Three years of experimental data from different Sea Water Reverse Osmosis (SWRO) desalination plants were used to identify the parameter of the model. They calculated the parameter for permeate flow decrements of 25% and 20%.

(13) $$A_{n}=t^{m}$$

where *m* is a parameter with values between −0.035 and −0.041 [26] related to permeate flow decline of 20% and 25%, respectively, and *t* is the operating time in days.

Zhu et al. [27] also proposed a model (Equation (14)) to predict the coefficient *A*. This involves an exponential equation, but in this case, a hollow fiber membrane was utilized (Dupont™ B-10, Wilmington, DE, USA) during one year of operating time. This correlation is not based on experiments, but on model-based simulation: variable feed pressure (6.28–7.09 MPa), constant feedwater concentration and temperature (35,000 mg/L and 27 ${}^{{}^{\circ}}$C, respectively). Belkacem et al. [65] used the Zhu model in terms of membrane resistance increase. The membrane used was the BW30LE-440 Filmtec™ (Midland, MI, USA) in a two-stage desalination plant with re-circulation during one year of operation.

(14) $$A_{n}=A_{0}\cdot e^{\left(\frac{-t}{\tau }\right)}$$

where τ is a correlative parameter, and the value was 328 under the aforementioned operating conditions.

Abbas et al. [28] (Equation (15)) proposed a model to determine the variation of the normalized average water permeability coefficient $A_{n}=A/A_{0}$, where $A_{0}$ is the initial average water permeability coefficient. It was an exponential equation depending on three parameters and time, and the utilized membrane was the BW30-400 Filmtec™. Five years of operating data were used for the parameter identification. The feedwater temperature was between 28 and 30 ${}^{{}^{\circ}}$C, the concentration being in a range of 2540–2870 mg/L, and the feed pressure was around 1200 kPa.

(15) $$A_{n}=\alpha \cdot e^{\left(\frac{\beta }{t+\gamma }\right)}$$

where α=0.68, β=79 and γ=201.1 for the aforementioned membrane and operating conditions.

A forth model was proposed by Ruiz-García et al. [29] (Equation (16)). They include the parameter $k_{\mathrm{fp}}$ in the model and gave specific information about the behavior of the performance decline in the long term. They proposed a two-stage pattern in the decline of *A* in RO systems: an initial Stage I, where a more pronounced decline than Stage II was shown. This is mainly due to membrane compaction, irreversible fouling (strongly adherent films) and $k_{\mathrm{fp}}$. Stage II is related to a gradual decrease mostly due to irreversible fouling and the frequency and efficiency of the Chemical Cleaning (CC). The model described the mentioned stages by the superposition of two exponential functions. The used about 3300 operating days of a full-scale brackish water reverse osmosis (BWRO) desalination plant to fit the parameters of the model. They got three equations, one related to maximum values of the normalized water permeability coefficient ($A_{n}$) (Post-Chemical Cleaning (Post-CC)), average and minimum values (Pre-Chemical Cleaning (Pre-CC)). This allowed obtaining equations to estimate a range of values for the coefficient $A_{n}$ in time.

(16) $$A_{n}=\delta_{1}\cdot e^{-\frac{t}{\tau_{1}}\cdot k_{\mathrm{fp}}}+\delta_{2}\cdot e^{-\frac{t}{\tau_{2}}\cdot k_{\mathrm{fp}}}$$

**Figure 3:**
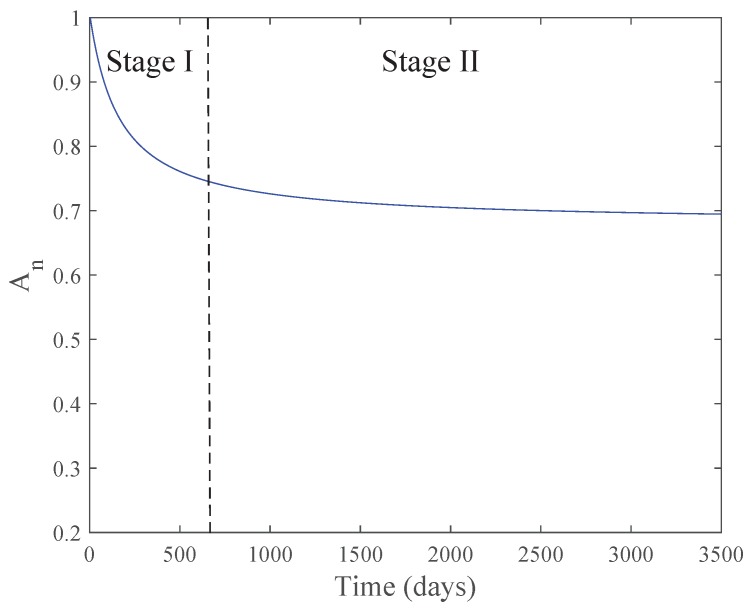
Schematic presentation of the two stages in $A_{n}$ decline. (I) initial more pronounced drop due to compaction and irreversible fouling; (II) gradual decline mainly caused by irreversible fouling [29].

The first exponential function is dependent on three parameters ($\delta_{1}$, $\tau_{1}$ and $k_{\mathrm{fp}}$) and is related to the behavior in Stage I (Figure 3), while the second is dependent on two parameters ($\delta_{2}$, $\tau_{2}$ and $k_{\mathrm{fp}}$) and is more related to Stage II (Figure 3). The first function gets closer to zero as Stage I ends. The δ are related to the weight of each exponential: the lower $\delta_{1}$ is and the higher $\delta_{2}$ is, the higher $A_{n}$ is when the desalination plant is stabilized. τ concerns the decline in each stage (i.e., how fast is the irreversible effects (mainly fouling) affecting performance): the larger the value, the more constant is the function. Generally, the higher $k_{\mathrm{fp}}$ results in a faster decline of $A_{n}$ in Stages I and II. They also carried out a comparison between the different models by using their experimental data.

## 4. Conclusions and Perspective of Future

The analysis of the different techniques, parameters, indexes and models that have been developed to date in the characterization and evaluation of RO membrane fouling potential reveals the existence of gaps in effective methods for the characterization and evaluation of fouling. It seems that the efforts made to advance our knowledge have turned out to be ineffective in terms of the mitigation and control of membrane fouling due to gaps in effective methods for the characterization and evaluation of fouling. The task of developing reliable fouling prediction tools is extremely important for the desalination industry, since fouling is one of the main causes of performance decrease in full-scale RO desalination plants. There are different fouling rates that have been developed and used in this field, but there remains much work to be done to improve these methods, indices and evaluation parameters. Among the weaknesses or deficiencies observed in the current methods of fouling assessment are the following:

(a) Most conventional indexes, SDI and MFI are not appropriate.
(b) There are very few studies about indices or parameters applied directly to spiral wound membranes and feedwater with high salinity. Most of the studies are applied at the laboratory scale with well-controlled operating conditions, flat membrane systems and at low salinity. However, it is preferable for fouling potential to be determined with RO membranes and under operating conditions similar to those of full-scale desalination plants.
(c) Currently, the effect of Cake-Enhanced Osmotic Pressure (CEOP) has not been taken extensively into account in measuring fouling potential. However, CEOP can contribute to a significant loss of performance, even more than the hydraulic resistance brought about by cake formation.

The aforementioned prediction models are based on long-term data of full-scale RO desalination plants under full-scale operating conditions. Unfortunately, these models do not take into consideration important features of membranes such as the spacer geometry. The efficiency and frequency of chemical cleanings, which play an important role in the performance of this process, should also be considered in these models.

## Figures and Tables

**Table 1:** Summary of various methods, indices and parameters used in fouling evaluation (adapted from [38,45,56]). SDI, Silt Density Index; MF, Microfiltration; MFI, Modified Fouling Index; UF, Ultrafiltration; CFS, Cross-Flow Sampler; RO, Reverse Osmosis; MMAS, Multiple Membrane Array System; DFI, Dimensionless Fouling Index; CEOP, Cake-Enhanced Osmotic Pressure.

Methods, Indices and Parameters	Characteristics	Equation	Comments
SDI (1995, [30])	- Membrane: MF 0.45 μm (flat sheet) - Foulant: particulate matter - Operation mode: dead-end and constant pressure - Fouling mechanisms: none - Test: time vs. volume	$SDI=\frac{1-\frac{t_{i}}{t_{f}}}{t}\cdot 100$	Disadvantages: SDI is a standardized method (ASTM D4189), but empirical, and it is not based on fouling mechanisms. It is not related to foulant concentration in feedwater. It does not take into account the temperature or variation in membrane resistance.
MFI (J.C. Schippers and J. Verdouw, 1980 [31])	- Membrane: MF of 0.45 μm (flat sheet) - Foulant: particulate matter - Operating mode: dead-end and constant pressure - Fouling mechanisms: cake filtration - Test: t/V vs. *V* (i.e., each 30 s)	$MFI=\frac{\mu \cdot I}{2\cdot \Delta p\cdot A^{2}}$	Characteristics: $MFI_{0.45}$ is an improved version of SDI and is related to cake filtration theory. The fouling index *I* is obtained from the slope of the lineal region of the graph t/V vs. *V* (filtrated volume). Disadvantages: It is not very accurate as foulant agents with a diameter less than 0.45 μm pass across the membrane.
$SDI^{+}$ (A. Alhadini et al., 2011 [44])	- Membrane: MF 0.45 μm (flat sheet) - Foulant: particulate matter - Operation mode: dead-end and constant pressure - Fouling mechanisms: none - Test: time vs. volume	—	Characteristics: $SDI^{+}$ is a normalization of SDI taking into consideration the variation of temperature, pressure and membrane resistance. Different fouling mechanisms could be assumed based on line charts and parameters’ calculation. Disadvantages: It is not very accurate as foulant agents with a diameter less than 0.45 μm pass across the membrane.
$SDI_{v}$ (A. Alhadini et al., 2011 [44])	- Membrane: MF 0.45 μm (flat sheet) - Foulant: particulate matter - Operation mode: dead-end and constant pressure - Fouling mechanisms: none - Test: time vs. volume	$SDI_{v}=\frac{100\cdot A_{M0}}{V_{f0}}\left(1-\frac{t_{1}}{t_{2}}\right)$	Characteristics: $SDI_{v}$ showed a more linear relationship with foulant concentration in feedwater than standard SDI. Besides, it is independent of testing parameters, such as temperature and pressure, and less sensitive to membrane resistance. Disadvantages: It is not very accurate as foulant agents with a diameter less than 0.45 μm pass across the membrane.
$MFI-UF_{\mathrm{const}.\;\mathrm{pressure}}$ (S.F.E. Booerlage et al., 1997 [57])	- Membrane: UF (hollow fiber, 13 kDa) - Foulant: particulate matter - Operating mode: dead-end and constant pressure. - Fouling mechanisms: cake filtration - Test: t/V vs. *V* or Δt/ΔV vs. *V* (i.e., each 10 s)	$MFI-UF=\frac{\mu \cdot I}{2\cdot \Delta p\cdot A^{2}}$ $MFI-UF=\frac{\mu \cdot \alpha_{0}\cdot C_{b}\cdot \Delta p^{\omega }}{2\cdot \Delta p\cdot A^{2}}$	Characteristics: UF membrane is used instead of MF, so colloidal fouling can be detected. $\alpha_{0}$ is a constant, ω the compressibility factor of the cake and $C_{b}$ the concentration of particles in the feedwater. Disadvantages: $MFI-UF_{\mathrm{const}.\;\mathrm{pressure}}$ is not able to show fouling behavior in constant flow precesses. Twenty hours are required to obtain a measurement, and the method to obtain the deposition factor is tedious. Although the UF membrane used in the tests is capable of retaining particles and colloidal matter, it is not efficient enough to retain organic matter.
$MFI-UF_{\mathrm{const}.\;\mathrm{flux}}$ (S.F.E. Boerlage et al., 2004 [46])	- Membrane: UF (flat sheet, 10–200 kDa) - Foulant: colloids - Operating mode: dead-end and constant flux. - Fouling mechanisms: cake filtration - Test: Δp vs. *t* or Δt/ΔV vs. *V*	$MFI-UF_{\mathrm{const}.\;\mathrm{flux}}=\frac{\mu_{20^{o}}\cdot c^{I}}{2\cdot \Delta p_{0}\cdot A_{0}^{2}}$ $MFI-UF=\frac{\mu \cdot \alpha_{0}\cdot C_{b}\cdot \Delta p^{\omega }}{2\cdot \Delta p\cdot A^{2}}$	Characteristics: The operating mode is constant flow as happens in the majority of actual RO processes. The fouling index *I* is obtained from the slope of the graph NDP (Net Driven Pressure) vs. filtration time. $\Delta p_{0}$ is the standard pressure (2 bar). Disadvantages: The test is performed under conditions of accelerated flow that do not allow representation of the behavior of fouling to flows of 20–30 L/m${}^{2}$h. As with $MFI-UF_{\mathrm{const}.\;\mathrm{pressure}}$, the deposition of particles is considered through a deposition factor, and although through the UF, it is possible to retain particulate matter and colloids, it is not enough to retain the organic matter present in the feed. Despite the improvements of $MFI-UF_{\mathrm{const}.\;\mathrm{flux}}$, the measurement cannot be simulated in cross-flow.
NF−MFI (S. Khirani et al., 2006 [42])	- Membrane: NF - Foulant: organic matter - Operating mode: dead-end and constant pressure. - Fouling mechanisms: cake filtration - Test: t/(V/A) vs. V/A	$MFI-NF=\frac{\mu \cdot I}{2\cdot \Delta p\cdot A^{2}}$	Characteristics: The test tries to take into consideration the organic matter in the feedwater. Disadvantages: The test is carried out under constant pressure, and the deposition factor of particles in cross-flow is not considered. The total retention of organic matter is not achieved in this procedure.
CFS−MFI (S.S. Adham and A.G. Fane, 2008 [48])	- Membrane: MF - Foulant: particulate matter - Operating mode: cross-flow and dead-end (separated)/constant pressure. - Fouling mechanisms: cake filtration - Test: t/V vs. *V*	$CFS-MFI=\frac{\mu \cdot \alpha \cdot C_{b}}{2\cdot \Delta p\cdot A^{2}}=\frac{\mu \cdot I}{2\cdot \Delta p\cdot A^{2}}$	Characteristics: This index incorporates the hydrodynamic behavior of the cross-flow in the measurement of the fouling index. CFS allow small particle to pass across the MF membrane to be deposited on the MF membrane located in MFI in dead-end flow. Disadvantages: Discontinued operating mode.
CFS−MFI (M.A. Javeed et al., 2009 [49])	- Membrane: MF - Foulant: particulate matter - Operating mode: cross-flow and dead-end/constant pressure - Fouling mechanisms: cake filtration - Test: t/V vs. *V*	$CFS-MFI=\frac{\eta_{20^{{}^{\circ}}C}\cdot \alpha \cdot C_{b}}{2\cdot \Delta p\cdot A^{2}}=\frac{\eta_{20^{{}^{\circ}}C}\cdot I}{2\cdot \Delta p\cdot A^{2}}$	Characteristics: CFS−MFI is measured in continuous mode. Disadvantages: It uses the same MF membrane as in MFI, and the operating mode is at constant pressure.
$CFS-MFI_{\mathrm{UF}}$ (L.N. Sim et al., 2011 [58])	- Membrane: MF and UF - Foulant: colloids - Operating mode: cross-flow and dead-end, constant flow - Fouling mechanisms: cake filtration - Test: Δp vs. *t*	$CFS-MFI_{\mathrm{UF}}=\frac{\mu \cdot I^{\prime }}{2\cdot \Delta p\cdot A^{2}}$	Characteristics: This index takes into account the hydrodynamic effect of cross-flow and the deposition factor. $I^{\prime }$ is the modified resistivity of the cake. $CFS-MFI_{UF}$ can be a more precise method to determine the effect of fouling agents on the RO process. The method is easy due to its short time of filtration.
CFI (J. Choi et al., 2009 [53])	- Membrane: MF and NF - Foulant: - Operating mode: constant pressure - Fouling mechanisms: - Test: t/V vs. *V*	$CFI=\frac{\mu \cdot \alpha \cdot C_{b}}{2\cdot \Delta p\cdot A^{2}}=\frac{\mu \cdot I}{2\cdot \Delta p\cdot A^{2}}$ $CFI=w_{1}\cdot M_{1}+w_{2}\cdot M_{2}+w_{3}\cdot M_{3}+w_{4}$	Characteristics: It is a combination of various indices, denoted as MFI−HL (using a Hydrophilic MF membrane), MFI−HP (using a MF Hydrophobic membrane) and MFI−UF (using a hydrophilic UF membrane). This test tries to take into consideration all types of foulant agents using different membranes. $M_{1}$ is the value of MFI−HL; $M_{2}$ is the value of MFI−HP; and $M_{3}$ is the value of MFI−UF. The weighting factors $w_{1}$, $w_{2}$, $w_{3}$ and $w_{4}$ depend on the characteristics of the membrane. Disadvantages: The method is difficult since it requires different types of membranes, and the procedure to obtain CFI is very tedious. In addition, the fouling index is still measured under constant pressure conditions.
MMAS (Y. Yu et al., 2010 [54])	- Membrane: MF, UF and NF - Foulant: particulate, colloids and organic matter - Operating mode: dead-end flow and constant pressure - Fouling mechanisms : - Test: t/V vs. *V*	—	Characteristics: MF, UF and NF membranes are connected in series for simultaneous separation of target foulants. This index was shown to be precise and selective in the prediction of the fouling potential of different feedwaters. Disadvantages: The method is not simple since it requires different types of membranes to determine the particle- MFI, colloid-MFI and organic-MFI. Furthermore, the fouling indices are still measured under constant pressure conditions.
DFI (M. W. Naceur, 2014 [55])	- Membrane: MF of 0.45 μm (flat sheet) - Foulant: particulate matter - Operating mode: dead-end and constant pressure - Fouling mechanisms: cake filtration - Test: t/V vs. *V*	$DFI=\frac{R_{m}^{2}}{2\cdot r\cdot C}$	Characteristics: The experimental procedure is similar to MFI. By introducing the equation of Ruth in the model, the authors obtained a dimensionless fouling index, which is a simple linear equation. Disadvantages: Experimental work to validate DFI was not carried out, so the accuracy of this index has not been validated.
“Normalized Fouling Rate” (NFR) (H.R. Rabie et al. 2001 [21])	- Membrane: - Foulant: - Operating mode: - Fouling mechanisms: - Test: $t/V_{s}$ vs. $V_{s}$	—	Characteristics: This method is used to analyze data from a pilot plant in a large-scale facility. NFR is the curve of the graph $t/V_{s}$ vs. $V_{s}$, where $V_{s}$ is the specific volume (the volume collected per unit area and per NDP in time *t*). Disadvantages: It cannot be used as a fouling potential indicator of feedwater.
$k_{\mathrm{fp}}$ (L. Song et al. 2004 [59])	- Membrane: UF and RO - Foulant: colloids - Operating mode: constant pressure - Fouling mechanisms: cake filtration - Test: *J* vs. *t*	$k_{\mathrm{fp}}=\frac{R_{t}^{*}-R_{0}^{*}}{v_{t}}$	Characteristics: This normalization method has the objective of eliminating the effects of different operating parameters in the determination of the fouling rate. In this way, the fouling potential of feed water can be compared on a fair basis. Disadvantages: One of its results indicates that the fouling potential of large colloidal particles increases as the operating pressure increases. This is mainly due to the compressibility effect of the cake, which is strongly related to the nature of the colloid.
Membrane Fouling Simulator (MFS) (J.S.Vrowenvelder et al. 2006 [60])		—	MFS uses the same membrane materials as spiral-wound RO/NF membrane, with the same dimensions and hydrodynamic behavior, and is equipped with a visor. Suitable for in situ observations in real time, non-destructive observations and parameters such as pressure drop can be monitored. It is mainly used as a biofouling monitor [61]. Disadvantages: There is no instant response of the fouling potential.
Feed Fouling Monitor (FFM) (A.H. Taheri et al., 2013 [17])		—	This technique uses a UF membrane to predict the increase of transmembrane pressure at constant fluxes in the presence of colloidal fouling. This prediction includes the developing hydraulic resistance and the CEOP components. Disadvantages: Lack of extension of this monitoring and modeling approach to real-world foulants and a full-scale RO desalination plant.
Feed Fouling Monitor-Salt Tracer Response (FFM-STRT) (A.H. Taheri et al., 2015 [16])		—	This method uses the FFM including an STRT to measure the development of concentration polarization in estimating (CEOP) the contribution. Foulants studied were humic acid and colloidal silica Disadvantages: There is no instant response of the fouling potential.
